# The complete mitochondrial genome of *Agriotes fuscicollis* Miwa, 1928 (Coleoptera: Elateridae)

**DOI:** 10.1080/23802359.2022.2119822

**Published:** 2022-09-15

**Authors:** Linxiao Wei, Tong Zhou, Jingru Ke, Yunzhu Sun, Feiping Zhang

**Affiliations:** a College of Forestry, Fujian Agriculture and Forestry University, Fuzhou City, China; b Key Laboratory of Integrated Pest Management in Ecological Forests, Fujian Province University, Fujian Agriculture and Forestry University, Fuzhou City, China; c College of Life Science, Fujian Agriculture and Forestry University, Fuzhou, China

**Keywords:** Complete mitochondrial genome, *Agriotes fuscicollis*, phylogenetic analysis

## Abstract

In this study, we sequenced the complete mitochondrial genome of *Agriotes fuscicollis* Miwa, 1928 (Coleoptera: Elateridae). The results showed that the length of complete mitochondrial genome was 15,866 bp with 26.8% GC content, containing 39.6% A, 33.5% T, 16.8% C, 10% G. There were 13 protein-coding genes (PCGs), 22 transfer RNA genes, and 2 ribosomal RNA genes. Phylogenetic analysis showed that *A. fuscicollis* was closely related to *Cryptalaus larvatus*, *Cryptalaus yamato*, *Pyrophorus divergens* and *Ignelater luminosus*. The complete mitogenome of *A. fuscicollis* would contribute to the study of the phylogeny and evolution of Elateridae.

*Agriotes fuscicollis* Miwa, 1928 (Coleoptera: Elateridae) is one of the important underground pests (Xue et al. 1985). They are distributed all over China and are common in the northwest of China. The adults have a preference for wheat leaves and newly decayed grass weeds, and have a strong tendency to slightly wilting weeds (Liu et al. 1988; Traugott et al. 2015). The larvae mainly damage the buds and seeds of wheat, corn and other crops, causing crops to wither and die, which can result in considerable economic losses (Liu et al. 1988; Pan and Ma 2006). However, no genetic evolutionary analysis of *A. fuscicollis* has been reported yet. This research adopted the Maximum-likelihood tree model to explore the phylogenetic relationship of *A. fuscicollis*. Illumina sequencing was employed to determine the whole mitogenome of *A. fuscicollis.* These achievements of the study would enrich correlative research content of the genetic evolution of *A. fuscicollis*, which is of great significance for the control of this insect.

The *A. fuscicollis* specimens were collected from Hongwei, Fujian Province, China (118°57′15″E, 26°09′09″N) using the snares with sexual attractants and deposited at the Fujian Agriculture and Forestry University (https://lxy.fafu.edu.cn, Songqing Wu, dabinyang@126.com) under the voucher number KJ-202101. The total genomic DNA of *A. fuscicollis* was extracted adopting TruSeq DNA Sample Prep Kit (Vazyme, CHN). We confirmed the DNA concentration and quality by NanoDrop 2000 (TFS, USA) and used Illumina Hiseq 2500 (Illumina, USA) to complete DNA sequencing. After filtrating, we got a total of 849,296 clean reads from the 53,874,634 raw reads. By adopting metaSPAdes and MitoZ, the clean reads were assembled (Meng et al. 2019). In addition, we adopted tRNAscan software to calculate tRNA genes (Chan and Lowe 2019). The whole mitogenome sequence of *A. fuscicollis* has been recorded in GenBank with accession number OM161961.

The whole mitogenome of *A. fuscicollis* was 15,866 bp in size. GC content of this whole genome was 26.8% (G = 10%, T = 33.5%, A = 39.6%, C = 16.8%). Besides, the whole mitogenome of *A. fuscicollis* was composed of 22 tRNA genes, 13 protein-coding genes (PCGs) and 2 rRNA genes. 3,714 amino acids were encoded by 13 PCGs which were 11,142 bp in all. 12 PCGs (*COX1, COX2, COX3, ND5, ND4L, ND4, ND3, ND2, ND6, ATP6, ATP8, CYTB*) adopted ATN as a start codon and the PCG (*ND1*) started with codon TTG. Among the 13 PCGs, 8 PCGs (*COX1, ATP6, ATP8, ND4L, ND2, ND6, ND5,* and *ND4*) ended with codon TAA, 3 PCGs (*ND1, CYTB* and *ND3*) used TAG as a stop codon, the PCG (*COX2*) stopped with codon CAT, and one PCG (*COX3*) stopped with codon ATA. Nine PCGs (*ND2, ND3, ND6, ATP6, ATP8, CYTB, COX1, COX2, COX3*) were clockwise coding, but four PCGs (*ND5, ND4L, ND4, ND1*) were counterclockwise coding. The stop codons of the PCGs (*COX2, COX3, ND4* and *ND5*) were unusual. The length of the rrnL and rrnS genes were determined to be 1,317 and 750 bp.

For purposes of determining the phylogenetic status of *A. fuscicollis*, the phylogenetic tree consisted of relevant 18 species of Coleoptera and one species of Lepidoptera adopting MEGAX software, which the *Anoplophora horsfieldi* was used as an out-group. By analyzing the evolutionary tree, we could learn that *A. fuscicollis* and 10 Elateridae that included Agrypninae, Prosterninae, Denticollinae, Semiotinae and Melanotinae formed a monophyletic group. In the research, a sister group to the clade of Agrypninae included the monophyletic Elateridae species. In addition, *A. fuscicollis* constituted a paraphyletic group with *Cryptalaus larvatus*, *Cryptalaus yamato*, *Pyrophorus divergens* and *Ignelater luminosus* (Figure 1). The complete mitogenome of *A. fuscicollis* can be conducive to the study of phylogeny evolution as well as the prevention of Elateridae. By this way, we can use the control methods of neighboring species in the evolutionary tree as experimental control of *A. fuscicollis*.

**Figure 1.**
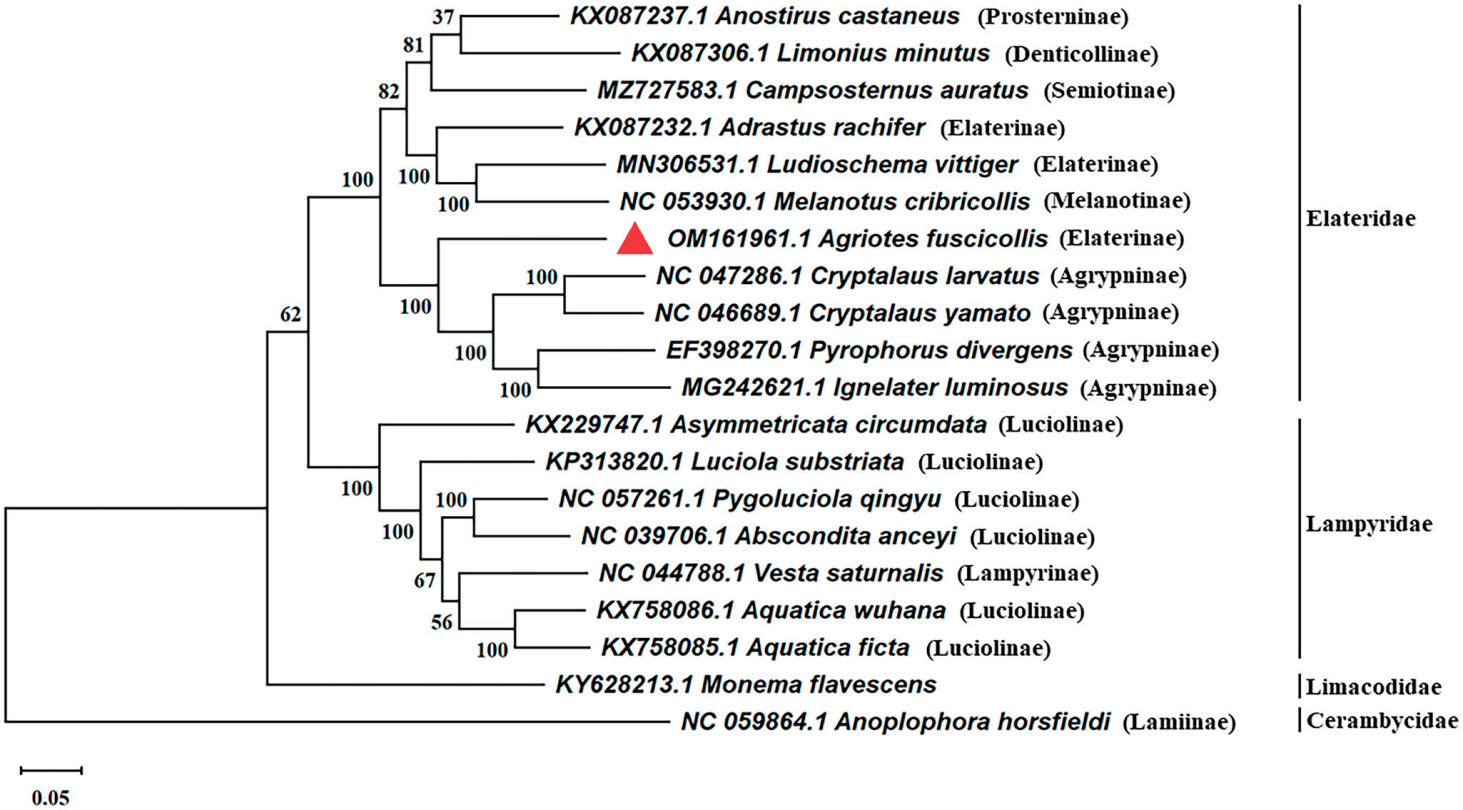
Maximum-likelihood tree of *Agriotes fuscicollis* Miwa and related 19 different species insects based on the mitochondrial genome. Numbers labeled on the branch are bootstrap values.

## Data Availability

The genome sequence data that support the findings of this study are openly available in GenBank of NCBI at https://www.ncbi.nlm.nih.gov under the assession no. OM161961. The associated BioProject, Bio-Sample and SRA number are PRJNA796305, SAMN24840756, and SRR17554247, respectively.

## References

[CIT0001] Chan PP, Lowe TM. 2019. tRNAscan-SE: searching for tRNA genes in genomic sequences. Methods Mol Biol. 1962:1–14. 31020551 10.1007/978-1-4939-9173-0_1 PMC6768409

[CIT0002] Liu CF, Yan J, Zhang XH. 1988. A method for rearing and observation on the life cycle of slender thorax Click Beetle (*Agriotes fuscicollis* Miwa). J Gansu Agric Univ. (2):51–55.

[CIT0003] Meng G, Li Y, Yang C, Liu S. 2019. MitoZ: a toolkit for animal mitochondrial genome assembly, annotation and visualization. Nucleic Acids Res. 47(11):e63. 30864657 10.1093/nar/gkz173 PMC6582343

[CIT0004] Pan T, Ma HP. 2006. Study on the occurrence regularity and control technology of *Agriotes fuscicollis* Miwa. Gansu Agricultural Science and Technology. (8):29–30.

[CIT0005] Traugott M, Benefer CM, Blackshaw RP, van Herk WG, Vernon RS. 2015. Biology, ecology, and control of elaterid beetles in agricultural land. Annu Rev Entomol. 60(1):313–334. 25341096 10.1146/annurev-ento-010814-021035

[CIT0006] Xue SZ, Zhang FQ, Ji Y, Gao JQ. 1985. The preliminary research on *Agriotes fuscicollis* Miwa. Shaanxi J Agric Sci. (3):9–11.

